# Optimization of Microwave Pre-Cooked Conditions for Gelatinization of Adzuki Bean

**DOI:** 10.3390/foods11020171

**Published:** 2022-01-10

**Authors:** Jianyou Zhang, Xuehua Xie, Lyu Zhang, Yiling Hong, Gaopeng Zhang, Fei Lyu

**Affiliations:** College of Food Science and Technology, Zhejiang University of Technology, Hangzhou 310014, China; zhjianyou@zjut.edu.cn (J.Z.); xxh_1996@163.com (X.X.); lyuzhang2022@126.com (L.Z.); food_safety2021@163.com (Y.H.); zhanggaopeng@zjut.edu.cn (G.Z.)

**Keywords:** pre-cooked, response surfaces, microwave gelatinization, starch structure

## Abstract

Pre-cooked adzuki beans (*Vigna angularis*), which looks like dried adzuki bean, is easily cooked and preserved. This study aimed to optimize the microwave pre-cooked conditions on adzuki beans by applying the response surface methodology. The results showed that soaking time has a significant effect on the gelatinization degree of adzuki beans according to microwave time. The most suitable gelatinization and the sensory scores were obtained with a soaking time of 7.8 h, a microwave power of 830 W, and microwave time of 92 s. The pre-cooked treatment had no significant effect (*p* > 0.05) on the protein, free amino acid, fat and starch content of adzuki bean products. The results of SEM and polarized light microscopy showed that the surface and center of starch were damaged after microwave treatment. XRD showed that microwave pre-cooking did not change the crystal structure of starch and maintained the original order of type A structure while reducing the relative starch crystallinity. FT-IR showed that the pre-cooked treatment did not produce new structure in adzuki bean starch, but the ratio of 1047/1022 cm^−1^ was slightly decreased, indicating that the starch crystallization area decreased relative to the amorphous area and the relative crystallinity decreased. The results of FTIR were consistent with X-ray diffraction results. Therefore, microwaves improved the gelatinization of adzuki beans and made the pre-cooked adzuki beans more suitable.

## 1. Introduction

Adzuki bean (*Vigna angularis*) belongs to the Leguminosae Papilionoideae family and the adzuki beans are most popular as compared to white, black and gray in northeast Asia [1]. The adzuki beans are mainly cultivated in China (670,000 ha), Japan (60,000 ha), South Korea (25,000 ha) and Taiwan (15,000 ha) [2]. The bean is commercially grown in the US, South America and India, as well as in New Zealand, Kongo and Angola. Adzuki beans are commonly used as food in the form of sprout boiled and as a drink such as tea. Some Asian cultures enjoy adzuki bean paste as a filling or topping for various kinds of waffles, pastries, baked buns, or biscuits. Adzuki beans are a good source of dietary fiber and minerals which include magnesium, potassium, and zinc [3]. It also contains large amounts of vitamin B1 that can assist in carbohydrate digestion [4]. The adzuki bean has a large consumer market in Asian countries. In China, adzuki bean is usually served as one of the popular foods on the table. In addition, adzuki beans also have the functions of nourishing the heart, nourishing the spleen and stomach, eliminating edema, dampness and clearing heat, and detoxification [5].

Nowadays, there is a novel trend cooking rice with beans and other grains due to the higher nutritional quality and dietary habits in some countries. Rice cooking with beans can improve the rough taste of beans and, on the other hand, can improve the human body lysine deficiency and improve the nutritional value [6]. The thing that makes Adzuki beans different from rice is the fact that they require long hours of soaking before cooking, usually 1–2 h [7]. If there is no soaking treatment, it will take long hours of cooking and does not attend to the growing consumer demand for fast preparation of food [8]. Moreover, the long time of cooking will reduce the rice and other grain’s textural and nutritional qualities. Therefore, it is necessary to study a pre-cooked method (before cooking) to reduce soaking time or make it possible to cook directly within a short time, so that the pre-cooked adzuki beans could be cooked together with rice or other grains to prepare varieties of mixed cereal products.

The microwave processing technology is widely used in chemistry due to its advantages of fast heating, space-saving and precise process control [9]. The microwave shows outstanding advantages and to take the advantage of microwave to heat because of the short heating time. It can ensure that the color and flavor of food can be retained to a large extent, especially in food thawing and drying [10]. Therefore, microwave processing may be an attractive alternative. It lays a certain theoretical foundation for microwave pretreatment of beans. Moreover, thermal processing does not involve the change of D-glucopyranose unit of starch polymer molecules, but usually only changes the accumulation and arrangement of starch polymer molecules as well as the overall structure of starch particles. Such changes may have a significant impact on starch properties, gelatinization, viscosity, and digestibility [11]. Therefore, microwave pre-gelatinization is of great significance for the research and improvement of starch granule structure changes during food processing and for controlling food quality [12].

Legume seeds before their consumption need to be processed to improve nutritional profile and decrease the non-nutritional factors [13]. Heat treatment such as cooking is the most common method of processing legumes, which mainly results in significant reductions in phytic acid, oligosaccharides, minerals, tannins and phenolic compounds content [14]. At the same time, anti-nutrients were reduced, providing greater α -amylase accessibility for starch digestion assays [15]. Some studies confirm that heat treatment significantly improves protein quality [16]. However, research on microwave pre-cooked adzuki beans are rarely studied in the world and limited study is available on structure of Adzuki bean starch changes during gelatinization. Our study effectively utilized the advantages of microwaves and deeply analyzed the changes of bean starch after microwave pre-cooked.

## 2. Materials and Methods

### 2.1. Beans

Dry beans (*Vigna angularis*) with a moisture content ≤ 10% were purchased from a local store in Hangzhou, China. The adzuki beans named Jihong NO.7, grow up in Ji Lin province, packed in plastic bags. Similar shape and size samples of adzuki beans were selected during this study.

### 2.2. Pre-Cooked Process

The pre-cooked processes included adzuki beans was soaking and microwaving, and then heat pump was used for drying set. The drying temperature to 55 °C for 4 h. The moisture content of adzuki beans at the end of drying can be less than 10%. The range of key control factors was roughly determined by pre-experiments with analysis by following the previous study [17]. We selected the soaking time, microwave power and microwave time as the key control factors in this research. First, single-factor experiments analyzed with microwave power 800 W, microwave time 80 s, the pre-gelatinization effect of selecting soaking time (2, 4, 6, 8, 10 h) were compared and dried at 55 °C for 4 h. Next, the pre-gelatinization effects of microwave power and time were studied. The microwave power was selected (600, 700, 800, 900, 1000 W), the soaking time was fixed at 4 h, and microwave time at 80 s. The microwave time was selected (70, 80, 90, 100, 110 s), with the soaking time at 4 h, and the microwave power at 800 W (Table 1). Each experiment was performed in triplicate. To evaluate the pre-gelatinization effect, the degree of gelatinization and the sensory score of adzuki beans treated by microwave were analyzed.

After the single-factor experiments the central composite design was used to optimize the microwave pre-gelatinization for adzuki beans. The moisture content of samples was kept controlled 8.20% ± 0.68% after the drying process. The design matrix with 20 experimental with five replicates of the midpoint is shown in Table 2. The comprehensive score of the gelatinized score (%) and the sensory evaluation of adzuki beans was taken as the response, *Y* and the evaluation index of microwave pre-gelatinization. The comprehensive score of the degree of gelatinization and the sensory evaluation of adzuki beans is the response value of the dependent variable for the 20 combinations of three independent variables (i.e., soaking time A, microwave power B and microwave time C) as independent variables.

### 2.3. Gelatinization Analysis

The gelatinization analysis determination of reducing sugar content by using 3,5-dinitrosalicylic acid method [14]. The degree of gelatinization was calculated by following equation:

Gelatinization degree α = (sample reducing sugar content-raw bean reducing sugar content)/fully gelatinized reducing sugar content × 100%

The sample means the uncooked adzuki beans after soaking, microwave pretreatment and heat pump drying.

### 2.4. Sensory Evaluation

Sensory parameters including color and appearance of pre-cooked adzuki beans prepared under different process conditions were determined by the previously described method [6]. The color depends on whether the color of samples is natural and shiny. The appearance depends on whether the overall shape of samples is uniform, and the cracks of miscellaneous beans are tiny or invisible. For the sensory evaluation, based on the principle of volunteering, we selected 10 volunteers, 5 males and 5 females from the college of food science and technology of the Zhejiang University of Technology. Sensory evaluation includes appearance and color, each accounting for 7 points and the final score is the sum of the two scores. The higher the score is when the color and appearance of the pre-ripened Adzuki beans are close to that of the commercially available adzuki beans. We have got the approval from the College of Food Science and Technology of Zhejiang University of Technology regarding the research ethics of the experiments.

### 2.5. The Comprehensive Score

This experiment is calculated by the method of standardization of dispersion to eliminate the effects of large units and small units (eliminating the dimension) [18]. The values normalized by the two indicators (gelatinization and sensory evaluation) are multiplied by their respective weight coefficients (0.5 and 0.5, discussed by the group), and the sum is the comprehensive score (*Y*) (showed by the following equation) and the highest score is 1 point. According to the formula, bring in the maximum and the minimum value of gelatinization degree and the sensory score.

Comprehensive score (*Y*) = (Gelatinization − 33.738)/(80.198 − 33.738) × 0.5 + (Sensory score − 7)/(11 − 7) × 0.5

In this formula, the 33.738 and 80.198 represent the maximum and minimum values of gelatinization degree, 0.5 as weight factor, 7 and 11 are the minimum and maximum values of sensory scores, respectively.

A second-order polynomial equation was then used to fit the measured, dependent variable (*Y*) as a function of the coded, independent variables (*Xi*). Regression analysis was performed for the experiment data and fitted to the empirical second order polynomial model, as shown in the following equation:

(1) $$Y=b0+\sum_{i=1}^{3}b_{i}X_{i}+\sum_{i=1}^{3}b_{ii}X_{i}^{2}+\sum_{i\;<\;j}^{3}b_{ij}X_{i}X_{j}$$

where *Y* is the response variable, *b0*, *bi*, *bii* and *bij* are the regression coefficients of variables for constant, linear, quadratic, and interaction regression terms, respectively; *Xi* and *Xj* are the coded values of the independent variables. The effects of three independent parameters soaking time (A) as *X_1_*, microwave power (B) as *X_2_* and microwave time (C) as *X_3_* on the comprehensive score (*Y*) were investigated using central composite design.

The fitted polynomial equation is expressed as surface and contour plots to visualize the relationship between the response and experimental levels of each factor and to deduce the optimum conditions [19].

### 2.6. Optimal Condition Verification

Three validation experiments were performed under these conditions to consider the reliability of the model.

### 2.7. Evaluation under Optimal Conditions

After three validation experiments, the gelatinization degree and sensory score were evaluated of the comprehensive score under the final pre-gelatinization condition.

#### 2.7.1. Texture Profile Analysis

The pre-cooked of the raw adzuki beans was carried out according to the above-mentioned optimum processing conditions. The cooked rice was prepared, and the raw adzuki beans without soaking and the pre-cooked adzuki beans were cooked at the same time, temperature and pressure according to the preparation process of the rice for textural measurements. The textural measurements were performed with a texture analyzer (TA-XT plus, Stable Micro Systems, Surrey, UK) fitted with a cylindrical probe (R 36). Texture profile analysis (TPA) tests were performed with a deformation level of 75% and crosshead speed was 5.0 mm/s. The textural parameters of adzuki beans were hardness (maximum force required to compress the sample in the first compression cycle), cohesiveness (area under the second peak/area under the first peak), springiness (the second compression distance/the original compression distance), gumminess (hardness × cohesiveness) and chewiness (hardness × cohesiveness × springiness). Take six adzuki beans at a time, measure them in parallel five times, remove the maximum and minimum values, and take the average as the result [20].

#### 2.7.2. Proximate Composition Analysis

The content of moisture, protein, fat, starch and amino acid were determined according to AOAC, respectively.

#### 2.7.3. Polarized Light Optical Microscopy and Scanning Electron Microscopy

The adzuki bean starch (protein content ≤ 0.23%) was isolated and purified from fresh adzuki beans and microwave pre-cooked adzuki beans for subsequent research.

The native starch granules and amylose single crystals were observed in suspension between crossed with an optical microscope equipped with the camera. The microstructure of freeze-dried starch gels was studied with a scanning electron microscope (SEM, Gemini 500, Carl Zeiss Co., Ltd., Oberkochen, Germany) Starch Gels were prepared according to the method explained and placed in a freeze dryer (CHRIST, Alpha 4–2 LD Plus, Marin Christ Co., Ltd., Osterode, Germany). Cross-sections of gel samples were attached to aluminum stubs using double-sided adhesive tape and coated with gold using a sputter coater (Q150R- ES, Quorum Technologies Co., Ltd., East Sussex, England). Samples were observed with SEM at an accelerating voltage of 2 kV and magnification of × 100.

#### 2.7.4. Infrared Transmission (FT-IR)

Structural investigations were carried out by using an infrared transmission (FT-IR) Digilab Excalibur FTS 3000 Mx spectrometer. IR spectra were recorded in the wavenumber range of 3700–700 cm^−1^, with a resolution of 4 cm^−1^. The samples were measured in the form of KBr pellets with a sample/KBr ratio 2/200 mg.

#### 2.7.5. X-ray Diffraction (XRD)

For the determination of XRD patterns, the X’ Pert Pro powder diffractometer (PNAlytical) was used. The incident X-ray monochromatic beam (λ CuKα = 1.54 Å) was monitored by a photomultiplier. The rotating anode device was operated at 40 kV and 40 mA. Scattering was detected in the range defined by the scattering vector q [q = 4 sin (θ)/λ, with λ being the wavelength and θ the scattering angle] 0.04–2.70 nm^−1^.

All analyses were conducted in triplicate and the average values were reported. In addition, the moisture content of all samples is 10% when equilibrated.

### 2.8. Data Analysis

Response surface methodology was applied to the experimental data using a commercial statistical package, Design-Expert version 8.05 (Statease Inc., Minneapolis, MN, USA). A polynomial equation was fitted to the data to obtain a regression equation. Statistical significance of the terms in the regression equation was examined. Response surface plots were generated with the same software. Through the subsequent verification experiments and the theoretical value comparison analysis of the model, and the optimal pre-cooked condition for adzuki bean is also used to calculate the relative error.

## 3. Results and Discussion

### 3.1. Optimum Process Conditions for Microwave Pre-Gelatinization

#### 3.1.1. Determining Levels of Independent Variables for Microwave Pre-Gelatinization

**Figure 1:**
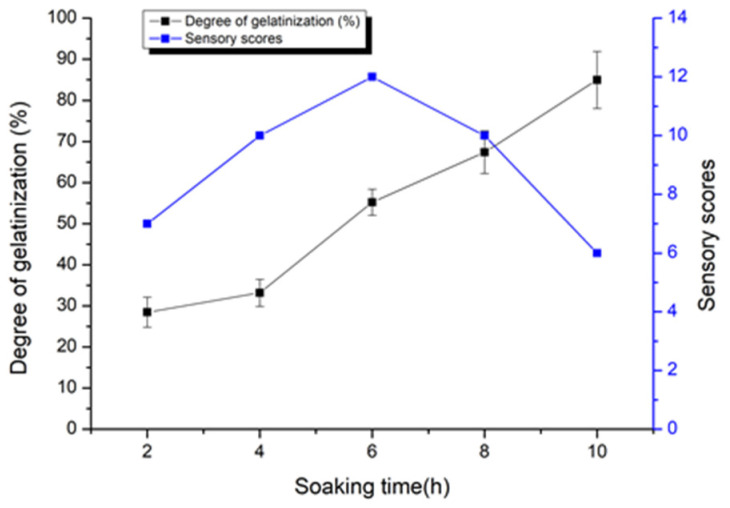
Typical changes in the degree of gelatinization and sensory scores of the soaking time.

The simple variable method was introduced in the experiments to find the relationship between gelatinization degree and sensory score with microwave processing parameters. As shown in Figure 1, the degree of gelatinization of the adzuki bean starch rose with the increasing soaking time. When the soaking time is 10 h, the gelatinization degree is the highest. This is because adzuki beans can absorb much water that expand it rapidly in the microwave process [21]. If the water molecules do not enter the crystallization zone of the starch molecules, the starch is difficult to gelatinize. The crystallinity, gelatinization and swelling of starch are related to amylopectin molecules [22]. However, the sensory scores of samples soaking 6 h are better than others. If the soaking time is too short, the adzuki beans do not absorb more water, which influences the adverse effect on gelatinization. Therefore, the optimal soaking time was found as 6 h during the study.

**Figure 2:**
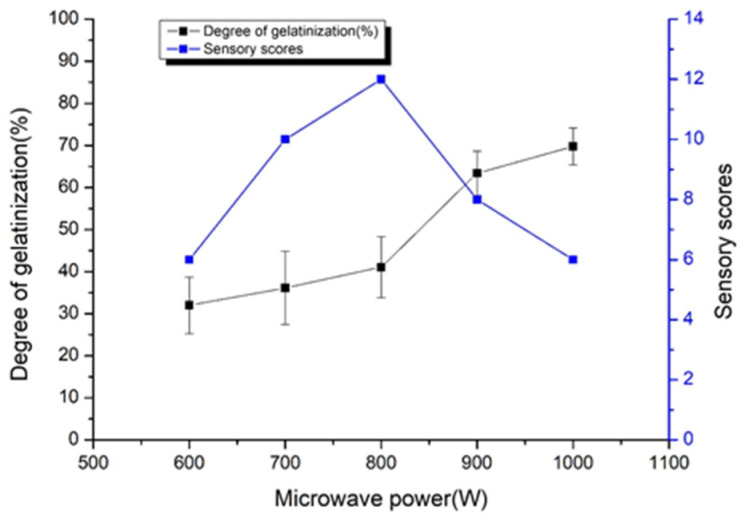
Changes in the degree of gelatinization and sensory scores of adzuki beans pre-cooked of different microwave power.

It can be seen from Figure 2 that as the microwave power was increased, the gelatinization degree of adzuki beans gradually increased. When the microwave power is small, the thermal effect and the vibration effect was not strong that resulting in a small degree of gelatinization of the adzuki beans starch. With the increase of microwave power, melting occurred in the crystallization zone and the hydrogen bond between starch molecules was broken, hydrophilic groups were exposed that improved the degree of gelatinization. The previous study proved that the higher the power, the more serious the damage, resulting in a greater degree of gelatinization [23,24]. The sensory score increased first and then decreased with the extension of microwave power. When the microwave power is 1000 W, the degree of gelatinization is the highest (69.75%), It also has a great influence on the color and appearance when the microwave power is too high. Therefore, suitable results were found with 800 W microwave power.

**Figure 3:**
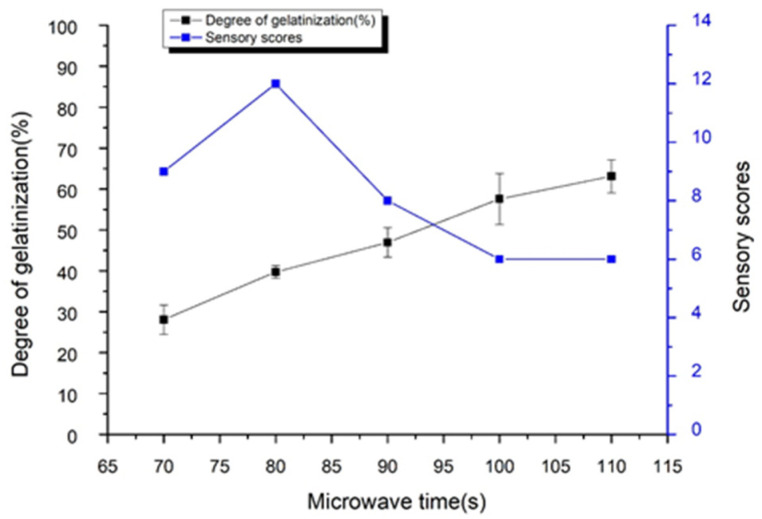
Changes in degree of gelatinization and sensory scores of adzuki beans pre-cooked of different microwave time.

The Figure 3 showed that as the microwave time increased, the gelatinization degree of adzuki beans gradually increased. This is because the strong vibrational motion of the polar molecules due to the application of microwave energy [22] accelerating the gelatinization of starch molecules. The sensory score increased first and then decreased with the extension of microwave time. When the microwave time was 110 s, the gelatinization degree was the highest (63.12%), which influenced on the color of adzuki beans. When the microwave time was set to 120 s, the adzuki beans were burnt. Therefore, the microwave time is optimally 80 s.

#### 3.1.2. Optimization of Process Conditions

The response surface test design was carried out based on the single factor test results and the results was presented in Table 2. Table 2 showed that the experimentally measured and the regression model predicted dependent variables. The comprehensive score of the degree of gelatinization and the sensory evaluation of adzuki beans is the response value of the dependent variable for the 20 combinations of three independent variables (i.e., soaking time A, microwave power B and microwave time C as independent variables). Design-Expert version 8.05 is used to analyze the data in Table 2. The results are as followed Table 3. According to Table 3, it was evident that the quadratic term coefficients (B2), and cross-product coefficients (AB, AC, BC) were not significant (*p* > 0.05). By performing quadratic multiple regression fitting on the data in the table, multiple regression equations between various factors and response values can be established:

*Y*1 = −98.46 + 3.50 A + 0.07 B + 0.90 C (2)

*Y*2 = −92.95 + 1.56 A + 0.09 B + 1.39 C − 0.013 AC − 0.09 A2 (3)

*Y* = −15.70 + 0.31 A + 0.011 B + 0.22 C − 0.022 A2 − 1.18767 E − 003 C2 (4)

*Y*2: Sensory evaluation *Y*1: Degree of gelatinization.

From the analysis of variance in Table 3, the *p*-value of the constructed quadratic regression model (comprehensive score) results is 0.0003 < 0.001, which indicates that the model is extremely significant; the *p*-value of the missing formula is 0.2319 > 0.05, and the surface model is not significant, which is compared with the actual fit. In addition, Table 4 also showed that the regression coefficients of the intercept, linear, and interaction terms of the model and the significance of each coefficient were determined using *p*-value. The *p*-value was used as a tool to check the significance of each coefficient and the smaller the *p*-value was, the more significant the corresponding coefficient was [25]. The primary item A soaking time (*p* < 0.0001) of the regression model was extremely significant. The primary item C microwave time (*p* = 0.0041) and B (*p* = 0.0310) had significant effects and there is no significant difference was found in these three items. In the Table 3, The C2 effect is extremely significant, the A2 effect is significant while the B2 effect is not significant.

From the analysis of variance in the Table 3 (results of sensory score), the primary item C microwave time (*p* = 0.5851) is not significant. However, B (*p* = 0.0275) microwave power has a greater impact on the sensory score compared to A soaking time (*p* = 0.0275) that both had identical significant effects. The interaction of the three items is also not significant in the Table 3. In the Table 3, the B2 and C2 effect is significant but the A2 effect was not significant.

Inverification of the optimal process it was found when the gelatinization degree of the pre-gelatinization treatment is more than 72%, the degree of gelatinization similar to that of rice can be achieved after adzuki beans re-cooking throughout the study. However, the degree of gelatinization was higher than 85% which became too soft and had poor color and palatability. Moreover, because of energy conservation, the time of microwaves during the pre-gelatinization process should be reduced. Considering the actual needs, the above process conditions are adjusted to determine the actual optimal microwave and ripe process conditions: soaking time 7.8 h, microwave power 830 W, microwave time 92 s, comprehensive score 0.82. Three validation experiments were performed under these conditions to consider the reliability of the model. The gelatinization was 74.67, the sensory score was 10and the comprehensive score was 0.82 (in Table 4). It showed that the optimization model is reliable and the optimization scheme is effective.

### 3.2. Composition of Untreated Adzuki Beans and Pre-Cooked Adzuki Beans

#### 3.2.1. Nutrient Analysis

The experimental data of untreated adzuki beans and pre-cooked adzuki beans are compared. The proximate composition of adzuki beans is shown in Table 5. The decrease in protein content is due to the loss of some water-soluble proteins and soluble sugars dissolved in water during the soaking process [26]. In addition, the content of fat is higher than that of the raw adzuki beans which may be due to starch gelatinization during the microwave heating, where the amylose and part of the fat complex disintegrate. However, overall, the main components of microwave pre-cooked adzuki beans have not changed compared with the untreated ones.

It can be seen from the Table 6 that threonine (Thr), valine (Val), isoleucine (Ile), leucine (Leu), phenylalanine (Phe), lysine (Lys) are among the detected amino acids are essential amino acids required by the human body [27]. The total amount of amino acids in the pre-cooked adzuki beans is lower than that of the raw mung beans, which is the same as the change in the protein in the table. This may be due to the dissolution of some water-soluble proteins during the pre-curing treatment, resulting in a decrease in the amino acid content [28].

#### 3.2.2. Texture Analysis

First, the evaluation of hardness is defined as the strength of the sample extruded by the tooth [29]. According to Table 7, the hardness of the pre-cooked adzuki beans and the rice was similar to that of the rice. However, the hardness of the native adzuki beans is much higher than that of the rice and the pre-cooked adzuki beans. The decrease of hardness may be due to the occurrence of claps caused by microwave drying [30]. Microwave drying can cause changes in the structure of starch inside cells and the decomposition of the intermediate thin layer of cells makes it easy to separate, which can promote the infiltration and migration of water during cooking [31]. The adhesiveness reflects the relative displacement of linear molecules such as amylose and the stickiness between the teeth during chewing. The adhesiveness of native adzuki beans was similar to the rice [32], but the pre-cooked adzuki beans are less adhesive. The springiness is defined as the ratio of the deformed sample to its original state when the pressure is removed [33]. The pre-cooked adzuki beans and the native adzuki beans have lower springiness than the rice. This is because the size of the adzuki bean particle is larger than the rice. Further the sample recovery height is larger before the second extrusion starts and the springiness value of pre-cooked adzuki beans is smaller than that of native adzuki beans. This is because the pre-cooked adzuki beans become softened after cooking and the particles become flat after the first extrusion. The chewiness is the energy that is required to chew a solid sample and the chewability value of the native adzuki beans is much greater than that of rice and pre-cooked adzuki beans. That indicates that the native adzuki beans are not easy to chew under the condition to cook with rice. So, the native adzuki beans not cooked together with the rice.

### 3.3. Starch Structure of Samples

#### SEM and Polarizing Microscope

**Figure 4:**
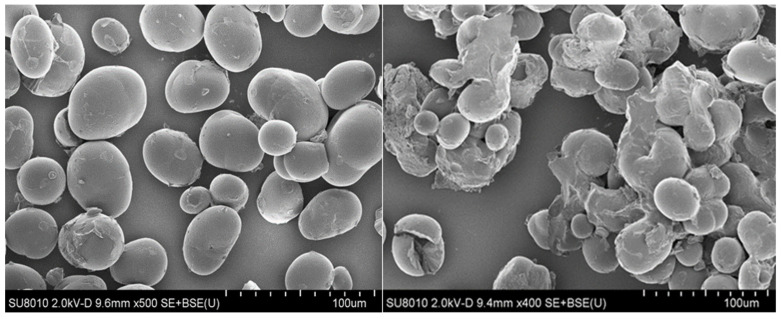
SEM pictures of adzuki bean starch at the magnification (100×). CK is shown on the left, MT is shown on the right.

The microstructure results was presented in Figure 4. According to Figure 4, SEM micrograph, the important impact of microwave on adzuki bean starch macrostructure of samples can be concluded. The native adzuki bean starch granules are spherical or ellipsoidal with obvious polarized cross and smooth surface [34]. These macrostructures almost appear to be intact treated with microwave, the starch granules appear concave and wrinkled. A small number of starch granules were broken and some starch granules were combined. This is because under high temperature, some starch particles adhere to each other or even fracture, the moisture in the original starch particles is removed, and the unevaporated part gathers on the surface of starch particles [35], the continuity and inhomogeneity of steam generation result was due to partial gelatinization [36].

**Figure 5:**
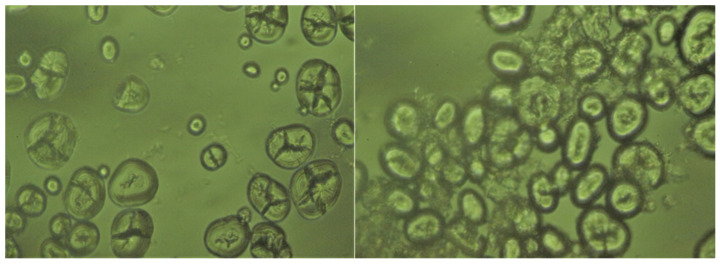
Polarizing microscope pictures of adzuki bean starch at the magnification (100×). CK is shown on the left, MT is shown on the right.

Figure 5 showed that the polarizing microscope of raw adzuki bean starch has obvious birefringence and the polarized cross is clear is the same in existing research results. However, at the same time, it can be seen that the individual starch particles have a deviation from the polarized cross. This may be due to the alkali action of the starch extraction process and the mechanical force of the agitation causing a slight loss of starch granules. The resulting polarizing microscope revealed that after microwave treatment and the polarization cross of some starch granules disappear that corresponds to the previous study [37].

### 3.4. Starch Structure

#### 3.4.1. X-ray Crystal Structure Analysis

Plant starch has three crystals of A-type, B- type and C- type [38] to the X-ray diffraction spectrum and the C-type is a mixed type of type A and type B adzuki bean starch is an A-type crystal and its XRD spectrum is characterized by strong diffraction peaks around 2θ (15°, 17°, 18° and 23°). There are strong diffraction peaks around 17°, 18° and 23° in which the diffraction peaks around 17° and 18° are connected double peaks, and there are weak diffraction peaks around 2θ = 20°. These results are in line same spectrum as the seed starch of other cereal crops that were found in a previous study [39].

By comparing the D value, 2θ and the strength in Table 8 it can be indicate that the pre-maturing (microwave treatment) did not change the main components of adzuki beans. The pre-maturing treatment did not change the ordered crystal structure of the untreated starch and remained the A-type structure.

It was calculated (Table 8) that the relative crystallinity of adzuki bean starch is 20.34% and the relative crystallinity of pre-cooked adzuki bean starch is 15.26%. The starch was relatively gelatinized and the amylose was dissolved while the relative crystallinity is reduced. The decrease in crystallinity of starch may be because the absorption of microwave electromagnetic energy by starch molecules becomes its kinetic energy during microwave cooking. Starch molecules collide and rub against each other during high-speed movement, further converting kinetic energy into heat energy. The heat caused the hydrogen bonds to break, destroying irregular regions, double helices and some crystalline regions [40]. During the gelatinization process, the content of the ordered structure of starch granules is greatly reduced and the amorphous content is rapidly increased that leading to an increase in the orderly crystal structure to the disordered arrangement of the amorphous state [41]. Eventually, the crystallization zone is destroying and the relative crystallinity is lowered, which is consistent with the FT-IR results.

#### 3.4.2. FT-IR Crystal Structure Analysis

**Figure 6:**
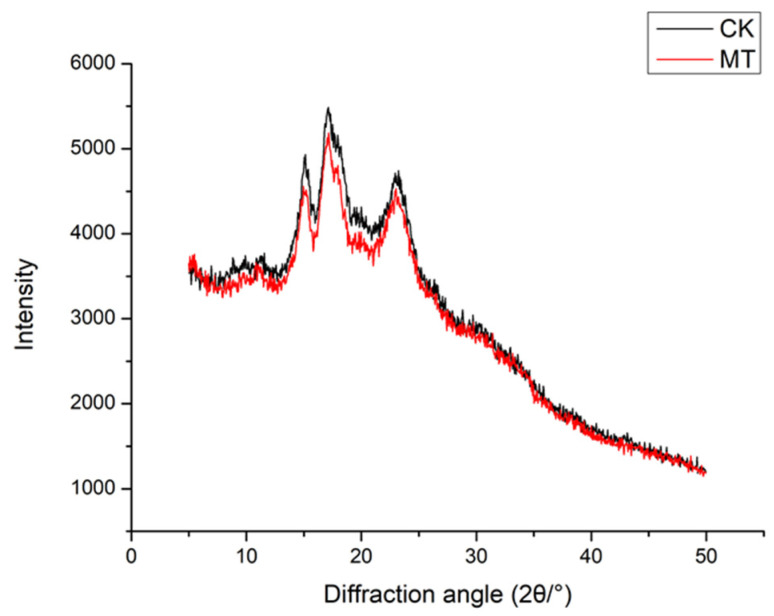
X-ray diffraction patterns of pre-cooked microwave adzuki beans starch samples as compared to untreated adzuki beans.

Comparing the infrared spectrum of native adzuki bean starch and pre-cooked (microwave) adzuki bean starch that can be seen in Figure 6 that the entire spectral range of 4000–500 cm^−1^ has a one-to-one correspondence of the absorption peaks of the spectrum and indicate that the pre-maturing treatment does not change the chemical bond composition of the starch molecules [42]. It does not produce new chemical structures.

The study by Habeych et al. [43] showed that the infrared absorption of the ordered structure of the starch crystallization zone at the wavenumber of 1047 cm^−1^, which reflects the number of starch crystallization zones. While 1022 cm^−1^ is the characteristic absorption of the starch amorphous zone. The ratio of absorbance at 1047–1022 cm^−1^ indicates the amount of starch crystallization zone relative to the amorphous zone.

It is calculated (Table 7) that the ratio of 1047/1022 cm^−1^ of adzuki bean starch is 1.040 and the ratio of 1047/1022 cm^−1^ of pre-gelatinized adzuki beans is 1.028. The decrease of the ratio of starch at 1047/1022 cm^−1^ indicates that the pre-cooked adzuki bean starch granules have a lower molecular order near the surface of the molecule which may be due to the dissociation of the double helix structure in the crystallization zone.

## 4. Conclusions

In the present study the response surface methodology was used to optimize the microwave pre-cooked conditions of adzuki beans. The most suitable gelatinization and the sensory score was obtained with a soaking time of 7.8 h, microwave power of 830 W, microwave time of 92 s. In addition, the microwave power of 830 W makes energy usage lower and it also benefited from industrialization. Soaking time has the most significant effect on the gelatinization degree of adzuki beans, followed by microwave time. After analysis of nutrients, the microwave did not destroy too much of the nutrients of adzuki beans. The texture results showed that the hardness and chewiness of pre-cooked adzuki beans were similar to those of rice. In addition, it reflects that the pre-cooked adzuki beans could cook with rice under the same steaming conditions of rice. Results of IR showed that the pre-cooked treatment did not change the main components of adzuki beans and the ratio of absorbance at 1047 to 1022 cm-1 reflected relative crystallinity. Although the orderly structure of the starch crystals did not change and was still the original ordered structure (A-type), the relative crystallinity of the starch decreased after treatment, which indicates that the starch structure became unstable. Therefore, microwaves improve the gelatinization of adzuki beans, making pre-cooked adzuki beans more suitable.

Further studies are needed to clarify the relative importance of hydro-thermal and dry-thermal stages in the pre-cooked (microwave) process and the MW-treated ability to improve the quality of adzuki beans products.

## Figures and Tables

**Table 1:** Single-factor experiments analysis.

Level	Factor		
	A Soaking Time /h	B Microwave Power /w	C Microwave Time /s
1	2	600	70
2	4	700	80
3	6	800	90
4	8	900	100
5	10	1000	110

**Table 2:** Results of gelatinization, sensory score and comprehensive score of adzuki beans treated with different factors in response surface experiment.

No.	A (h)	B (W)	C (s)	Gelatinization/%	Sensory Score	Comprehensive Score
1	6	800	90	66.63	11	0.85
2	6	800	90	64.61	10	0.67
3	6	800	70	43.68	7	0.15
4	4	700	100	51.62	8	0.36
5	6	800	90	64.51	11	0.79
6	8	700	100	65.75	9	0.60
7	6	1000	90	80.20	7	0.54
8	4	900	80	64.49	7	0.33
9	2	800	90	33.74	7	0.00
10	6	800	90	65.34	11	0.80
11	6	800	90	65.51	10	0.76
12	8	900	100	79.47	9	0.78
13	6	800	90	64.31	10	0.75
14	8	900	80	54.84	10	0.60
15	6	600	90	60.33	8	0.45
16	4	700	80	34.12	8	0.17
17	10	800	90	79.40	10	0.83
18	6	800	110	78.97	7	0.45
19	4	900	100	71.56	8	0.53
20	8	700	80	42.33	9	0.38

A, B and C represent treatment factors of soaking time, microwave power, and microwave time.

**Table 3:** Variance analysis of quadratic regression model for response surface experiment.

		Sum of Square	df	Mean-Square	F	*p*	Significant
Gelatinization	Module	2909.40	3	969.80	15.61	<0.0001	Significant
	A	782.93	1	782.93	12.60	0.0029	**
	B	844.74	1	844.74	13.59	0.0022	**
	C	1281.73	1	1281.73	20.63	0.0004	**
	Residual	932.73	15	62.14			
	Lack of fit	908.49	11	82.59	13.98	0.0106	Significant
	Error	23.63	4	5.91			
	Sum	3841.53	18				
Sensory score	Module	27.40	9	3.04	3.90	0.0003	Significant
	A	9.00	1	9.00	11.54	0.0275	*
	B	0.25	1	0.25	0.32	0.0079	**
	C	0.000	1	0.000	0.000	0.5851	N.S.
	AB	0.50	1	0.50	0.64	1.0000	N.S.
	AC	0.50	1	0.50	0.64	0.4440	N.S.
	BC	0.000	1	0.000	0.000	0.4440	N.S.
	A2	2.98	1	2.98	3.82	1.0000	N.S.
	B2	8.66	1	8.66	11.10	0.0088	**
	C2	12.61	1	12.61	16.17	0.0030	**
	Residual	7.02	9	0.78			
	Lack of fit	1.02	5	0.20	0.14	0.9747	N.S.
	Error	6.00	4	1.50			
	Sum	34.42	18				
Comprehensive score	Module	0.92	9	0.10	6.10	0.0003	Significant
	A	0.43	1	0.43	25.73	<0.0001	***
	B	0.053	1	0.053	3.15	0.0310	*
	C	0.12	1	0.12	7.07	0.0041	**
	AB	6.9 × 10^-4^	1	6.9 × 10^-4^	0.041	0.7765	N.S.
	AC	1.048 × 10^-6^	1	1.048 × 10^-6^	6.28 × 10^-5^	0.9912	N.S.
	BC	3.021 × 10^-5^	1	3.021 × 10^-5^	1.81 × 10^-3^	0.9525	N.S.
	A2	0.13	1	0.13	7.78	0.0009	**
	B2	0.066	1	0.066	3.96	0.0048	N.S.
	C2	0.25	1	0.25	15.03	0.0001	***
	Residual	0.15	9	0.017			
	Lack of fit	0.051	5	0.010	0.41	0.2319	N.S.
	Error	0.099	4	0.025			
	Sum	1.07	18				

A, B and C represent treatment factors of soaking time, microwave power, and microwave time, AB, AC and BC represent the interaction between soaking time and microwave power, soaking time and microwave time and microwave power and microwave time. * 0.01 < *p* < 0.05, ** 0.001 < *p* < 0.01, *** *p* < 0.001, N.S. represents not significant.

**Table 4:** Comparison of experimental data and model calculated value.

Treatments	The Experimental Data			Model Calculated Value			Relative Error		
	Gelatinization/%	Sensory Scores	Comprehensive Scores	Gelatinization/%	Sensory Scores	Comprehensive Scores	Gelatinization/%	Sensory Scores	Comprehensive Scores
C_1_	33.74	7	0.00	37.22	7.14	0.08	0.10 ± 1.81	0.02 ± 0.82	0.00 ± 0.065
C_2_	51.62	8	0.36	55.90	8.24	0.35	0.08 ± 0.92	0.03 ± 0.94	0.03 ± 0.032
C_3_	80.19	7	0.54	75.74	7.39	0.61	0.06 ± 2.02	0.06 ± 1.25	0.13 ± 0.003
C_4_	74.67	10	0.82	71.47	10.34	0.82	0.04 ± 2.11	0.03 ± 0.65	0.005 ± 0.012
C_5_	79.40	10	0.83	75.20	10.14	0.74	0.05 ± 1.09	0.01 ± 0.94	0.11 ± 0.007

C_1_: 2 h 800 W 90 s; C_2_: 4 h 700 W 100 s; 6 h; C_3_:1000 W 90 s; C_4_: 7.8 h 830 W 92 s; C_5_: 10 h 800 W 90 s.

**Table 5:** Changes of proximate composition of microwave precooked adzuki beans (Unit: g/100 g).

	Protein	Fat	Starch	Moisture
CK	20.48 ^a^ ± 0.35	0.72 ^a^ ± 0.20	49.17 ^a^ ± 0.16	10.78 ^a^ ± 0.15
MT	20.16 ^a^ ± 0.04	0.91 ^a^ ± 0.18	52.78 ^b^ ± 0.12	10.14 ^a^ ± 0.11
ST	20.22 ^a^ ± 0.15	0.80 ^a^ ± 0.11	49.42 ^a^ ± 0.21	19.05 ^b^ ± 0.13

CK, Untreated adzuki beans; MT, Microwave precooked adzuki beans; ST, adzuki beans after soaking. For each column, values followed by the different letters indicate significant differences (*p* < 0.05).

**Table 6:** The free amino acid content (mg/100 g) of untreated adzuki beans and microwave pre-cooked adzuki beans.

	Asp	Thr	Ser	Glu	Gly	Ala	Val	Met	Ile	Leu	Tyr	Phe	Lys	His	Arg	Pro	Sum
CK	1.98	0.44	0.80	2.72	0.91	1.01	1.01	0.37	0.91	1.55	0.35	0.91	1.11	0.53	1.91	0.60	17.12
MT	1.95	0.43	0.74	2.62	0.90	1.06	1.01	0.40	0.90	1.54	0.27	0.90	1.06	0.52	1.84	0.57	16.73

CK, Untreated adzuki beans; MT, Microwave pre-cooked adzuki beans.

**Table 7:** Analysis of texture properties of adzuki beans.

	Hardness/g	Adhesiveness/g.sec^−1^	Springiness	Cohesiveness/ss	Gumminess/s	Chewiness	Resilience
Rice	2108.89 ^b^ ± 18.56	66.98 ^a^ ± 3.27	0.61 ^a^ ± 0.21	0.51 ^a^ ± 0.07	890.12 ^a^ ± 6.54	638.16 ^b^ ± 5.11	0.21 ^a^ ± 0.07
CK	9640.76 ^a^ ± 23.54	63.17 ^b^ ± 5.18	0.56 ^b^ ± 0.18	0.38 ^b^ ± 0.09	3214.07 ^c^ ± 15.78	3075.39 ^a^ ± 14.64	0.21 ^a^ ± 0.09
MT	2246.67 ^b^ ± 17.68	59.73 ^c^ ± 5.34	0.47 ^c^ ± 0.31	0.21 ^c^ ± 0.05	592.77 ^b^ ± 8.67	526.54 ^b^ ± 6.77	0.097 ^b^ ± 0.03

CK, Untreated adzuki beans; MT, Microwave pre-cooked adzuki beans. For each column, values followed by the different letters indicate significant differences (*p* < 0.05).

**Table 8:** X-ray diffraction patterns and Fourier infrared spectrum of microwave pre-cooked adzuki beans starch.

Sample	X-ray					Fourier Infrared Spectrum		
	2θ/°	D	Strength	RC (%)	Crystal Type	Absorbance 1047 cm^−1^	Absorbance 1022 cm^−1^	Ratio (A(1047 cm^−1^/1022 cm^−1^))
CK	14.983	5.91	S	20.34	A-type	60.016	57.717	1.040
	16.895	5.24	S					
	23.143	3.84	S					
MT	15.070	5.87	S	15.26	A-type	68.912	67.066	1.028
	17.081	5.19	S					
	23.198	3.83	S					

CK, Untreated adzuki beans; MT, Microwave pre-cooked adzuki beans.

## Data Availability

Data is contained within the article.
